# Single Mild Traumatic Brain Injury Induces Persistent Disruption of the Blood-Brain Barrier, Neuroinflammation and Cognitive Decline in Hypertensive Rats

**DOI:** 10.3390/ijms20133223

**Published:** 2019-06-30

**Authors:** Nikolett Szarka, Luca Toth, Andras Czigler, Zoltan Kellermayer, Zoltan Ungvari, Krisztina Amrein, Endre Czeiter, Zsolt Kristof Bali, Sai Ambika Tadepalli, Matyas Wahr, Istvan Hernadi, Akos Koller, Andras Buki, Peter Toth

**Affiliations:** 1 Department of Neurosurgery and Szentagothai Research Center, University of Pecs, Medical School, Ret u. 2, H-7623 Pecs, Hungary; 2 Institute for Translational Medicine, Medical School, University of Pecs, Szigeti ut 12, H-7624 Pecs, Hungary; 3 Clinical Medicine Doctoral School, University of Szeged, Tisza Lajos krt. 109., H-6725 Szeged, Hungary; 4 Department of Immunology and Biotechnology, University of Pecs, Medical School, Szigeti ut 12, H-7624 Pecs, Hungary; 5 Reynolds Oklahoma Center on Aging, Donald W. Reynolds Department of Geriatric Medicine, University of Oklahoma Health Sciences Center, Oklahoma City, OK 73104, USA; 6 MTA-PTE Clinical Neuroscience MR Research Group, Ret u. 2, H-7623 Pecs, Hungary; 7 Translational Neuroscience Research Group, Szentagothai Research Center, University of Pecs, Ifjusag utja 20, H-7624 Pecs, Hungary; 8 Grastyan Translational Research Center, University of Pecs, Ifjusag utja 6, H-7624 Pecs, Hungary; 9 Cellular Neurobiology, Institute of Physiology, Medical School, University of Pecs, Szigeti ut 12, H-7624 Pecs, Hungary; 10 Department of Experimental Neurobiology, Faculty of Sciences, University of Pecs, Ifjusag utja 6, H-7624 Pecs, Hungary; 11 Department of Morphology and Physiology, Faculty of Health Sciences, Semmelweis University, Ulloi ut 26, H-1085 Budapest, Hungary; 12 Department of Physiology, New York Medical College, Valhalla, NY 10595, USA

**Keywords:** hypertension, vascular injury, mild brain trauma, BBB, cognitive dysfunction

## Abstract

Traumatic brain injury (TBI) induces blood-brain barrier (BBB) disruption, which contributes to secondary injury of brain tissue and development of chronic cognitive decline. However, single mild (m)TBI, the most frequent form of brain trauma disrupts the BBB only transiently. We hypothesized, that co-morbid conditions exacerbate persistent BBB disruption after mTBI leading to long term cognitive dysfunction. Since hypertension is the most important cerebrovascular risk factor in populations prone to mild brain trauma, we induced mTBI in normotensive Wistar and spontaneously hypertensive rats (SHR) and we assessed BBB permeability, extravasation of blood-borne substances, neuroinflammation and cognitive function two weeks after trauma. We found that mTBI induced a significant BBB disruption two weeks after trauma in SHRs but not in normotensive Wistar rats, which was associated with a significant accumulation of fibrin and increased neuronal expression of inflammatory cytokines TNFα, IL-1β and IL-6 in the cortex and hippocampus. SHRs showed impaired learning and memory two weeks after mild TBI, whereas cognitive function of normotensive Wistar rats remained intact. Future studies should establish the mechanisms through which hypertension and mild TBI interact to promote persistent BBB disruption, neuroinflammation and cognitive decline to provide neuroprotection and improve cognitive function in patients with mTBI.

## 1. Introduction

Traumatic brain injury (TBI) is a serious health problem worldwide resulting in significant chronic disabilities. After the mechanical force-induced primary brain injury, TBI initiates a variety of pathophysiological processes leading to secondary brain damage [1,2,3], which contributes to the development of long-term psychiatric problems and cognitive decline [2]. Disruption of the blood-brain barrier plays a central role in the secondary pathological processes (reviewed in [4]), leading to accumulation of blood-borne substances in the cerebral parenchyma, neuroinflammation and consequent decline in higher brain function. Mild TBI (mTBI) is the most frequent form of head trauma, typically affecting young athletes and the elderly population, who are prone to fall [5]. Interestingly, mild TBI is likely to induce blood-brain barrier (BBB) disruption, but it has only a transient effect [6,7,8]. A growing body of research has established that preexisting comorbid medical conditions exacerbate the deleterious effects of TBI (resulting in longer intensive care unit (ICU) stay and increased risk for complications) [9]. The most frequently identified comorbid condition in TBI is hypertension (in older individuals prevalence is about 38.8%) [10,11], which has been demonstrated to exacerbate disruption of the BBB in various pathological conditions. For example, hypertensive aged mice were found to exhibit increased permeability of the BBB, which is associated with neuroinflammation and cognitive decline of the animals [12]. Based on these, in the present study we tested the hypothesis that mild TBI induces persistent, long term disruption of the BBB in hypertensive rats, leading to persistent accumulation of blood borne substances in the brain parenchyma, neuroinflammation and cognitive decline.

## 2. Results

### 2.1. Mild TBI Induces Persistent Disruption of the Blood-Brain Barrier Which is Associated with Extravasation and Accumulation of Blood Borne Fibrin in Cerebral Tissue of Spontaneously Hypertensive Rats

**Figure 1:**
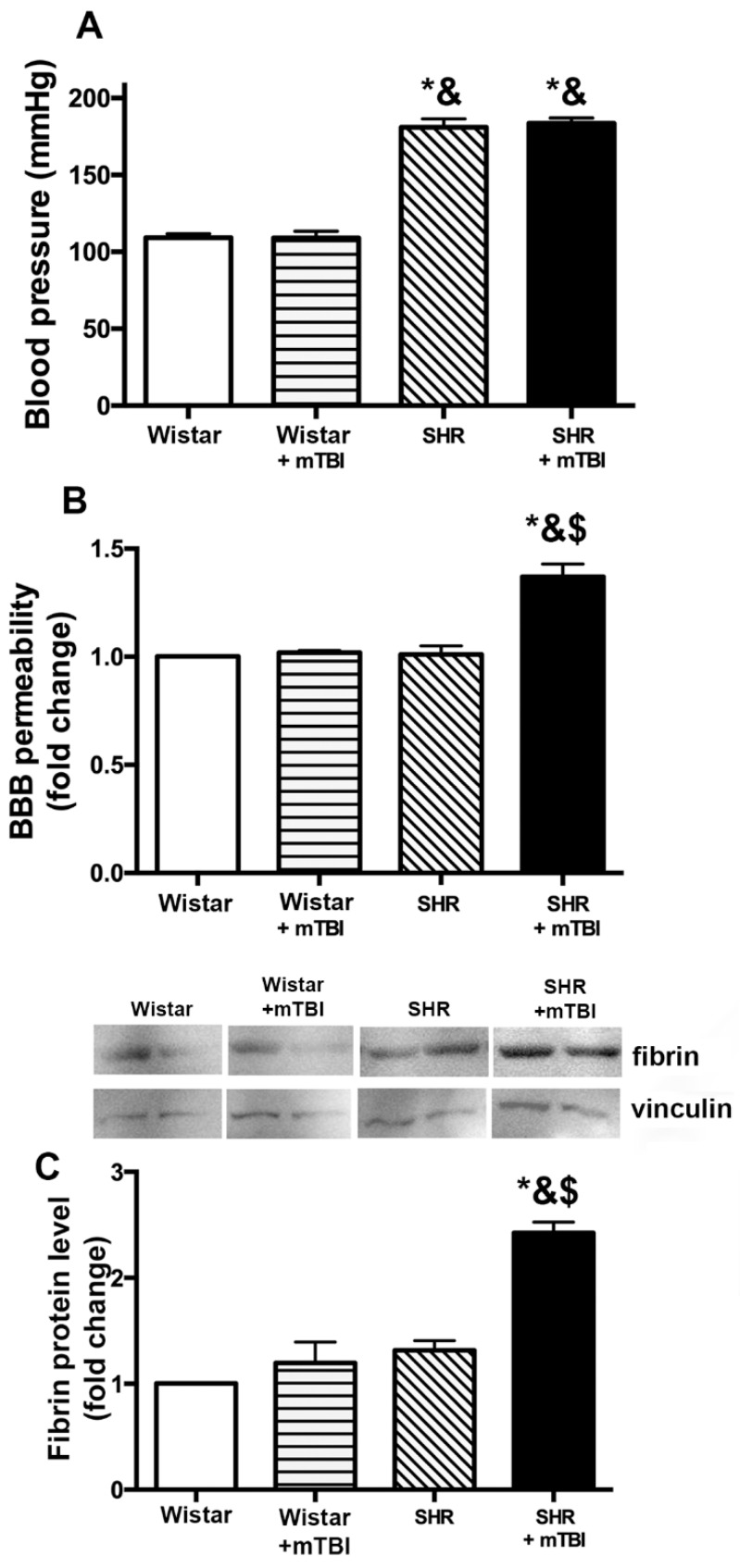
Mild traumatic brain injury (TBI) induces persistent disruption of the blood brain barrier and extravasation of blood borne substances in hypertensive rats. (**A**) shows blood pressure of Wistar rats and spontaneously hypertensive rats (SHR) with and without mild traumatic brain injury (mTBI) measured by the tail-cuff method. Data are means ± S.E.M. (*n* = 6 in each group) * *p* < 0.05 vs. Wistar, & *p* < 0.05 vs. Wistar + mTBI. (**B**) Summary data show blood brain barrier permeability indicated by extravasated Evans blue content of cerebral tissue (depicted as fold change compared to control) in sham operated Wistar rats and SHRs, and in rats two weeks after mTBI. Data are means ± S.E.M. (*n* = 6 in each group) * *p* < 0.05 vs. Wistar, & *p* < 0.05 vs. Wistar + mTBI, $ *p* < 0.05 vs. SHR. (**C**) One representative Western blot presents fibrin level in perfused cerebral tissue from Wistar and spontaneously hypertensive rats (SHR) with and without mild traumatic brain injury (mTBI) (showing two in each group) two weeks after trauma. Summary data depicts cerebral fibrin protein level in cortical tissue of the above groups of animals. Data are means ± S.E.M. (*n* = 6 in each group) * *p* < 0.05 vs. Wistar, & *p* < 0.05 vs. Wistar + mTBI.

Spontaneously hypertensive rats (SHR) had significantly higher blood pressure than normotensive Wistar rats (Figure 1A). Blood pressure was not affected by mTBI in either normotensive or hypertensive rats (Figure 1A). We found that mTBI led to a significant disruption of the blood-brain barrier detected two weeks after mTBI in hypertensive rats (*n* = 7), as shown by increased Evans blue content of cerebral tissue of these animals. No leakage of Evans blue dye could be observed in normotensive rats (*n* = 7), indicating intact BBB after mTBI (Figure 1B). We found that two weeks after mild TBI fibrin accumulated in cortical tissue of hypertensive rats (*n* = 6), which could not be observed in SHRs without mTBI (*n* = 6) or normotensive rats with and without mTBI (*n* = 6, in both groups) (Figure 1C).

### 2.2. Mild TBI Induces Persistent Neuroinflammation and Cognitive Decline in Spontaneously Hypertensive Rats

**Figure 2:**
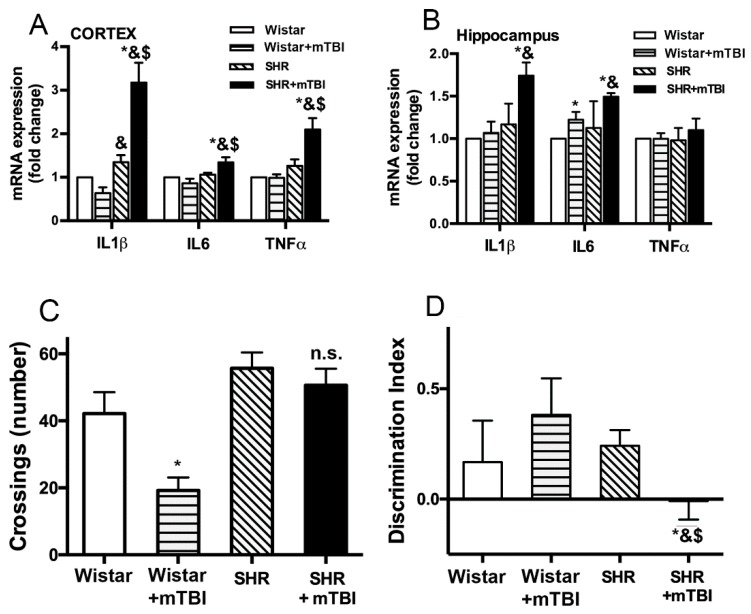
Mild TBI induces persistent neuroinflammation and cognitive decline in hypertensive rats. (**A**,**B**) mRNA expression of inflammatory cytokines IL1β, IL6 and TNFα in cortical (**A**) and hippocampal (**B**) tissue of sham operated normotensive Wistar rats and SHRs, and of animals two weeks after mild TBI, expressed as fold change compared to control. Data are means ± S.E.M. (*n* = 8 in each group) * *p* < 0.05 vs. Wistar, & *p* < 0.05 vs. Wistar + mTBI, $ *p* < 0.05 vs. SHR. (**C**) In a standard open field test normotensive Wistar animals showed attenuated exploratory activity (number of crossings) two weeks after mTBI (Wistar + mTBI) indicating habituation to the environment and intact locational memory function. In contrast, SHRs did not show habituation to the environment in the repeated open field test (OFT) session two weeks after mTBI (SHRmTBI), indicating impaired locational memory. Data are means ± S.E.M. (*n* = 15 in each group) * *p* < 0.05 vs. Wistar. (**D**) Intermediate-term declarative memory was tested two weeks after mTBI by the novel object recognition test. Discrimination index was not changed in normotensive Wistar rats two weeks after mTBI, but it was significantly decreased in SHRs, indicating impaired declarative memory of the animals. Data are means ± S.E.M. (*n* = 5 Wistar and *n* = 11 SHR) * *p* < 0.05 vs. Wistar, & *p* < 0.05 vs. Wistar + mTBI, $ *p* < 0.05 vs. SHR.

We found that expression of inflammatory cytokines IL-1β, IL-6 and TNFα was significantly (*p* < 0.05) increased in both cortical and hippocampal tissue of spontaneously hypertensive rats (*n* = 8) two weeks after mTBI compared to sham-operated SHRs (*n* = 8) and to normotensive Wistar rats with and without mTBI (*n* = 8 in both groups) (Figure 2A,B). 

We show that two weeks after trauma normotensive Wistar rats (*n* = 11) exhibited a significant (*p* < 0.05) decrease in the number of crossings (locomotor activity and exploration) in the open field arena, indicating habituation to the environment thus functioning learning and memory (Figure 2C). In contrast, SHRs (*n* = 11) did not show any changes in the number of crossings during the repeated session two weeks after mTBI, indicating a decline in their learning and memory functions (Figure 2C). We studied intermediate-term declarative memory by the novel object recognition test. We found that mTBI resulted in a significant (*p* < 0.05) decrease in the Discrimination Index (DI) in SHRs (*n* = 11) assessed two weeks after trauma indicating impaired memory function. Whereas DI was not changed in normotensive Wistar rats (*n* = 5), indicating preserved memory function (Figure 2D). 

## 3. Discussion

The salient finding of the present study is that mild TBI is sufficient to induce persistent disruption of the blood-brain barrier when pre-existing arterial hypertension is present, which is associated with the development of cognitive dysfunction (Figure 1 and Figure 2). These findings extend previous results that mild TBI-induced BBB disruption reaches a peak within hours and after 2 to 3 days post-injury [6,7,8], and has an important translational aspect to clinical setting: hypertensive patients should probably be assessed and treated after mild brain trauma differently than normotensive subjects. Our results also raise the possibility, that other co-morbidities known to increase the vulnerability of the cerebrovasculature to various insults, such as diabetes or obesity [13] would also exacerbate BBB disruption following mTBI and lead to cognitive decline. These possibilities should be verified by future studies. 

The mechanisms by which hypertension and mild TBI interact to promote persistent disruption of the BBB are likely multifaceted. Both TBI and hypertension have been shown to induce overproduction of reactive oxygen species [14,15,16]. Although mild TBI results in a transient increase in reactive oxygen species (ROS) generation [17], co-existing hypertension may exaggerate and extend production of ROS in the cerebrovasculature. ROS can directly damage tight junction proteins of the BBB [18], and activate redox sensitive matrix metalloproteinases [19], resulting in increased permeability of the barrier. Other proteases, like caspase, known to be activated in hypertension [20,21], may contribute to the process. These possibilities should be tested by future studies. 

We found that mild TBI leads to accumulation of fibrin in cerebral tissue of hypertensive rats (Figure 1). Previous studies suggested that the link between BBB disruption and neurological dysfunction is the accumulation of toxic blood borne substances in the brain parenchyma. For instance, in mice deficient of pericytes the injured BBB allows the deposition of fibrinogen in brain parenchyma [22], which is associated with activation of the monocyte/macrophage system [23], the increasing number of microglia and the increased production of ROS [24], further activation of proteases and production of inflammatory cytokines. According to this pathological process, we also found that two weeks after mild TBI production of inflammatory cytokines IL-1β, IL-6 and TNFα is significantly enhanced in the cortices and hippocampi of hypertensive rats compared to normotensive animals (Figure 2A,B), indicating mTBI-induced persistent neuroinflammation in SHRs. Inflammatory cytokines further promote BBB damage [25], probably producing a positive feed back loop and perpetuating the pathological process. There is most likely a causal link between mild TBI-induced persistent neuroinflammation and cognitive deficit (Figure 2C,D). This is supported by studies showing that in the mouse hippocampus chronic neuroinflammation structurally modifies axons and dendrites of neurons and dysregulates genes involved in regulation of neuronal function (such as Bdnf, Homer1, and Dlg4) [26,27], which may have a role in impaired synaptic plasticity and impaired cognitive function [26]. 

### Limitations of the Study

Despite the clear difference in cognitive function after mTBI in normotensive and hypertensive rats, due to within-subject study design, we cannot exclude that Wistar rats would exhibit better learning and memory function than those with mTBI. Brain trauma may induce an acute, transient increase in blood pressure, which can be recorded by continuous measurement of blood pressure, for example with telemetry. Lack of these data is a limitation of our studies. Hypertension has deleterious effects in addition to the direct effect of high blood pressure. Therefore, future studies should test whether treated hypertension exacerbates the effect of mild traumatic brain injury on BBB disruption, neuroinflammation and cognitive decline, as well. 

## 4. Materials and Methods 

### 4.1. Mild Traumatic Brain Injury (mTBI) in Normotensive and Hypertensive Rats

All procedures were approved by the Institutional Animal Use and Care Committee of the University of Pecs Medical School and the National Scientific Ethical Committee on Animal Experimentation, Hungary (nr: BAI/35/51-107/2016 on 25 October 2016) and carried out in accordance with the ARRIVE guidelines. Spontaneously hypertensive rats (SHR, male, 300–350 g, *n* = 30) and age-matched normotensive Wistar rats (Wistar, male, 300–350 g, *n* = 30) were purchased from Janvier Labs (Le Genest-Saint-Isle, France) and Toxi-Coop (Budapest, Hungary). Mild impact acceleration diffuse brain injury was induced by Marmarou’s weight drop model. Under isoflurane (2%) anesthesia the skull was exposed by a midline incision between the lambda and bregma and a steel disc was fixed with cement on the skull. A 450 g cylindrical weight from 250 mm was dropped to the disc causing mild diffuse traumatic brain injury to the animals. We aimed to apply “very mild” head injury, therefore we chose to follow the protocols of Singh et al., who demonstrated that weight dropped from 250 mm was sufficient to induce detectable histological, biomarker and behavioral changes in rats [28,29].

All animals survived the procedures. Blood pressure was measured 14 days after TBI before further experiments in all groups of animals using the tail cuff method, as described [30].

### 4.2. Behavioral Studies

#### 4.2.1. Open Field Test (OFT)

The open field test (OFT) was carried out in normotensive Wistar rats and SHRs (*n* = 15) before and two weeks after mTBI. The OFT arena is a black plywood box surrounded by walls. The floor of the arena is divided with gray lines to four by four equal squares. In each session, rats were allowed to explore the arena for 5 min. After each rat, the box was thoroughly cleaned using 20 *v*/*v* % ethanol. The sessions were recorded using a video camera (JVC super LoLux, JVC Kenwood Holdings Inc., Yokohama, Japan) and Ethovision XT10 software (Noldus, Wageningen, The Netherlands). During the sessions, the number of line crossings were registered as a measure of locomotor activity and exploration. The activity of rodents in an open field environment tends to decrease during repeated sessions. This habituation to the environment is considered as a measure of learning and memory ability of rodents [31], and learning and cognitive deficit is indicated by the lack of decrease in the number of crossings. 

#### 4.2.2. Novel Object Recognition Test (NOR) 

Declarative memory performance of the animals was assessed by the novel object recognition test (NOR) two weeks after mTBI in normotensive Wistar rats and SHRs. The NOR test consisted of two trials. Trial #1 (acquisition): two identical objects were placed in the arena, and the rats were allowed to explore them for 3 min. Then, the animals were removed from the arena. Trial #2 (recognition): after 30 min retention time, one of the objects from the 1st trial (familiar object) and a novel object were placed in the arena, and the behavior of the rats was recorded for 3 min. The animal was considered to be exploring an object when he was sniffing the object or put his nose close to it while facing the object. Time spent with the exploration of the novel (E_n_) and the familiar (E_f_) objects were compared by calculating a discrimination index (DI) using the following equation: DI = (E_n_ − E_f_)/(E_n_ + E_f_). The DI was a positive number if the novel object was observed for a longer time than the familiar object, and the DI was negative if the observation of the familiar object was longer than that of the novel object. DI was around zero if the two objects were observed for equally long time. 

### 4.3. Blood-Brain Barrier Disruption

Blood-brain barrier permeability was assessed in normotensive Wistar and spontaneously hypertensive rats (*n* = 7) two weeks after mild TBI by the Evans blue extravasation method. In brief, rats were anesthetized (isoflurane (2%)) and 2% Evans blue dye was administered intraperitoneally (7 mL/kg). The dye was allowed to circulate for 3 h. Then, the animals were transcardially perfused with PBS, decapitated, and the brains were removed. Right hemispheres were separated, weighed and homogenized in trichloroacetic acid (TCA) solution (50% TCA diluted in 0.9% saline). The samples were then centrifuged (12,000 rpm for 20 min), and absorbance of supernatants was measured at 630 nm with a spectrophotometer (BioTek Synergy HT Multi-Detection Microplate Reader (BioTek Instruments, Inc., Winooski, VT, USA).

### 4.4. Western Blotting

Cortical samples from normotensive Wistar rats and spontaneously hypertensive rats with and without mild TBI (*n* = 6) were homogenized on ice using 400 μL of modified RIPA buffer containing 10% methanol, protease and phosphatase inhibitors (1:200, 1:100 respectively). The lysate was agitated on 4 °C for 2 h, then centrifuged on maximum speed for 15 min. Lysates of samples were stored at −30 °C prior to Bradford’s protein assay. Following the protein concentration assay, the supernatant was suspended in Laemmli sample buffer (SigmaAldrich, Budapest, Hungary) and was heated to 100 °C for 15 min. Gel electrophoresis was carried out using mini-PROTEAN electrophoresis cell (Bio-Rad, Hercules, CA, USA), applying 80 and 120 V in stacking and developing regions. Blotting was carried out in a Novex Mini-Cell wet blotter (Invitrogen, Carlsbad, CA, USA) applying 450 mA for 2 h. Non-specific binding sites were blocked with 5% milk/TBS on room temperature for 2 h then incubated with primary antibodies overnight (Vinculin, #13901 Cell Signaling, Budapest, Hungary, and Fibrin GTX19079 GeneTex, Irvine, CA, USA) in 3% bovine serum albumin. Rinsing was performed with 0.1% Tween20/TBS, five times, then incubated with secondary HRP linked antibody on room temperature for 2 h. After series of rinsing of TBS-Tween, the blot was imaged using A Fujifilm CCD imager and densities were evaluated using ImageJ software [32]. 

### 4.5. Quantitative Real-Time RT-PCR 

Total RNA was isolated from cortex and hippocampus of rats from each group (*n* = 8) using NucleoSpin RNA (Macherey-Nagel GmbH, Düren, Germany) and purity and concentration were analyzed by NanoDrop. RNA was reverse-transcribed into cDNA with the High Capacity cDNA RT Kit (Life Technologies, Carlsbad, CA, USA). RT-PCR was run on an ABI-PRISM 7500 machine in duplicates using SensiFast SYBR Green (BioLine, London, UK). The following primers were used: il1, forward: 5’-GAG TCT GCA CAG TTC CCC AA-3’, reverse: 5’-ATG TCC CGA CCA TTG CTG TT-3’; il6, forward: 5’-CAC AAG TCC GGA GAG GAG AC-3’, reverse: 5’-GCC ATT GCA CAA CTC TTT TCT CA-3’; tnfα, forward: 5’-CAG CAA CTC CAG AAC ACC CT-3’, reverse: 5’-GGA GGG AGA TGT GTT GCC TC-3’; βactin, forward: 5’-GTA ACC CGT TGA ACC CCA TT-3’, reverse: 5’-CCA TCC AAT CGG TAG TAG CG-3’. Results are shown as percentage of housekeeping gene and expressed as fold change compared to control. Quantification was performed using the ΔΔ*C*T method.

### 4.6. Statistical Analysis

Data were analyzed by analysis of variance (ANOVA) and Tukey’s post hoc test. Behavioral experiments were evaluated by two-way mixed ANOVA with within-subject factor of pre- and post-injury measurements, and between-subject factor of groups (i.e., Wistar and SHR). A *p* value less than 0.05 was considered statistically significant. Data are expressed as mean ± S.E.M.

## 5. Conclusions

In conclusion, we show that in hypertensive rats, mild brain trauma is sufficient to cause a persistent disruption of the blood-brain barrier, which is associated with accumulation of toxic blood borne substances in the brain parenchyma, neuroinflammation and cognitive decline of the animals. We propose that hypertensive patients with mild TBI should be assessed differently than normotensive patients (by quantifying BBB function, cognitive function etc.), and the mechanisms by which hypertension and mild TBI interact should be established in order to selectively target BBB function and achieve neuroprotection in this patient population.

## Abbreviations

TBI	traumatic brain injury
mTBI	mild traumatic brain injury
BBB	blood-brain barrier
IL	interleukin
TNF	tumor necrosis factor
SHR	spontaneously hypertensive rat
ICU	intensive care unit
DI	discrimination index
ROS	reactive oxygen species
OFT	open field test
NOR	novel object recognition test
TCA	trichloroacetic acid

## References

[1] Schwarzmaier S.M., Terpolilli N.A., Dienel A., Gallozzi M., Schinzel R., Tegtmeier F., Plesnila N. (2014). Endothelial nitric oxide synthase mediates arteriolar vasodilatation after traumatic brain injury in mice. J. Neurotrauma.

[2] Sahuquillo J., Poca M.A., Amoros S. (2001). Current aspects of pathophysiology and cell dysfunction after severe head injury. Curr. Pharm. Des..

[3] Greve M.W., Zink B.J. (2009). Pathophysiology of traumatic brain injury. Mt. Sinai J. Med. New York.

[4] Alluri H., Wiggins-Dohlvik K., Davis M.L., Huang J.H., Tharakan B. (2015). Blood-brain barrier dysfunction following traumatic brain injury. Metab. Brain Dis..

[5] Gardner R.C., Yaffe K. (2015). Epidemiology of mild traumatic brain injury and neurodegenerative disease. Mol. Cell Neurosci..

[6] Rodriguez-Grande B., Ichkova A., Lemarchant S., Badaut J. (2017). Early to long-term alterations of cns barriers after traumatic brain injury: Considerations for drug development. Aaps. J..

[7] Li W., Watts L., Long J., Zhou W., Shen Q., Jiang Z., Li Y., Duong T.Q. (2016). Spatiotemporal changes in blood-brain barrier permeability, cerebral blood flow, t2 and diffusion following mild traumatic brain injury. Brain Res..

[8] Johnson V.E., Weber M.T., Xiao R., Cullen D.K., Meaney D.F., Stewart W., Smith D.H. (2018). Mechanical disruption of the blood-brain barrier following experimental concussion. Acta Neuropathol.

[9] Kumar R.G., Juengst S.B., Wang Z., Dams-O’Connor K., Dikmen S.S., O’Neil-Pirozzi T.M., Dahdah M.N., Hammond F.M., Felix E.R., Arenth P.M. (2018). Epidemiology of comorbid conditions among adults 50 years and older with traumatic brain injury. J. Head Trauma Rehabil..

[10] Centers for Disease Control and Prevention (2015). Report to Congress on Traumatic Brain Injury in the United States: Epidemiology and Rehabilitation.

[11] Thompson H.J., Dikmen S., Temkin N. (2012). Prevalence of comorbidity and its association with traumatic brain injury and outcomes in older adults. Res. Gerontol Nurs..

[12] Toth P., Tucsek Z., Sosnowska D., Gautam T., Mitschelen M., Tarantini S., Deak F., Koller A., Sonntag W.E., Csiszar A. (2013). Age-related autoregulatory dysfunction and cerebromicrovascular injury in mice with angiotensin ii-induced hypertension. J. Cereb Blood Flow Metab..

[13] Tucsek Z., Toth P., Sosnowska D., Gautam T., Mitschelen M., Koller A., Szalai G., Sonntag W.E., Ungvari Z., Csiszar A. (2013). Obesity in aging exacerbates blood-brain barrier disruption, neuroinflammation, and oxidative stress in the mouse hippocampus: Effects on expression of genes involved in beta-amyloid generation and alzheimer’s disease. J. Gerontol. A. Biol. Sci. Med. Sci..

[14] Marcano D.C., Bitner B.R., Berlin J.M., Jarjour J., Lee J.M., Jacob A., Fabian R.H., Kent T.A., Tour J.M. (2013). Design of poly(ethylene glycol)-functionalized hydrophilic carbon clusters for targeted therapy of cerebrovascular dysfunction in mild traumatic brain injury. J. Neurotrauma.

[15] Szarka N., Pabbidi M.R., Amrein K., Czeiter E., Berta G., Pohoczky K., Helyes Z., Ungvari Z., Koller A., Buki A. (2018). Traumatic brain injury impairs myogenic constriction of cerebral arteries: Role of mitochondria-derived h2o2 and trpv4-dependent activation of bkca channels. J. Neurotrauma.

[16] Pinto C.C., Silva K.C., Biswas S.K., Martins N., De Faria J.B., De Faria J.M. (2007). Arterial hypertension exacerbates oxidative stress in early diabetic retinopathy. Free Radic Res..

[17] Marklund N., Clausen F., Lewander T., Hillered L. (2001). Monitoring of reactive oxygen species production after traumatic brain injury in rats with microdialysis and the 4-hydroxybenzoic acid trapping method. J. Neurotrauma.

[18] Schreibelt G., Kooij G., Reijerkerk A., van Doorn R., Gringhuis S.I., van der Pol S., Weksler B.B., Romero I.A., Couraud P.O., Piontek J. (2007). Reactive oxygen species alter brain endothelial tight junction dynamics via rhoa, pi3 kinase, and pkb signaling. Faseb J. Off. Publ. Fed. Am. Soc. Exp. Biol..

[19] Haorah J., Ramirez S.H., Schall K., Smith D., Pandya R., Persidsky Y. (2007). Oxidative stress activates protein tyrosine kinase and matrix metalloproteinases leading to blood-brain barrier dysfunction. J. Neurochem..

[20] Sun H.J., Ren X.S., Xiong X.Q., Chen Y.Z., Zhao M.X., Wang J.J., Zhou Y.B., Han Y., Chen Q., Li Y.H. (2017). Nlrp3 inflammasome activation contributes to vsmc phenotypic transformation and proliferation in hypertension. Cell Death Dis..

[21] Wang Q., Cui Y., Lin N., Pang S. (2019). Correlation of cardiomyocyte apoptosis with duration of hypertension, severity of hypertension and caspase-3 expression in hypertensive rats. Exp. Ther. Med..

[22] Montagne A., Nikolakopoulou A.M., Zhao Z., Sagare A.P., Si G., Lazic D., Barnes S.R., Daianu M., Ramanathan A., Go A. (2018). Pericyte degeneration causes white matter dysfunction in the mouse central nervous system. Nat. Med..

[23] Fiala M., Liu Q.N., Sayre J., Pop V., Brahmandam V., Graves M.C., Vinters H.V. (2002). Cyclooxygenase-2-positive macrophages infiltrate the alzheimer’s disease brain and damage the blood-brain barrier. Eur. J. Clin. Investig..

[24] Carrano A., Hoozemans J.J., van der Vies S.M., van Horssen J., de Vries H.E., Rozemuller A.J. (2012). Neuroinflammation and blood-brain barrier changes in capillary amyloid angiopathy. Neuro-Degener. Dis..

[25] Rochfort K.D., Cummins P.M. (2015). The blood-brain barrier endothelium: A target for pro-inflammatory cytokines. Biochem. Soc. Trans..

[26] Cortese G.P., Burger C. (2017). Neuroinflammatory challenges compromise neuronal function in the aging brain: Postoperative cognitive delirium and alzheimer’s disease. Behav. Brain Res..

[27] Jaworski T., Lechat B., Demedts D., Gielis L., Devijver H., Borghgraef P., Duimel H., Verheyen F., Kugler S., Van Leuven F. (2011). Dendritic degeneration, neurovascular defects, and inflammation precede neuronal loss in a mouse model for tau-mediated neurodegeneration. Am. J. Pathol..

[28] Singh K., Trivedi R., Devi M.M., Tripathi R.P., Khushu S. (2016). Longitudinal changes in the dti measures, anti-gfap expression and levels of serum inflammatory cytokines following mild traumatic brain injury. Exp. Neurol..

[29] Singh K., Trivedi R., Haridas S., Manda K., Khushu S. (2016). Study of neurometabolic and behavioral alterations in rodent model of mild traumatic brain injury: A pilot study. Nmr Biomed..

[30] Szarka N., Amrein K., Horvath P., Ivic I., Czeiter E., Buki A., Koller A., Toth P. (2017). Hypertension-induced enhanced myogenic constriction of cerebral arteries is preserved after traumatic brain injury. J. Neurotrauma.

[31] Muller U., Cristina N., Li Z.W., Wolfer D.P., Lipp H.P., Rulicke T., Brandner S., Aguzzi A., Weissmann C. (1994). Behavioral and anatomical deficits in mice homozygous for a modified beta-amyloid precursor protein gene. Cell.

[32] Tarjanyi O., Berta G., Harci A., Bacsa E.B., Stark B., Pap M., Szeberenyi J., Setalo G. (2013). The role of src protein in the process formation of pc12 cells induced by the proteasome inhibitor mg-132. Neurochem. Int..

